# CXCR2 is critical for dsRNA-induced lung injury: relevance to viral lung infection

**DOI:** 10.1186/1476-9255-2-4

**Published:** 2005-05-28

**Authors:** Vedang A Londhe, John A Belperio, Michael P Keane, Marie D Burdick, Ying Ying Xue, Robert M Strieter

**Affiliations:** 1 Department of Pediatrics, David Geffen School of Medicine at UCLA, Los Angeles, CA, USA; 2 Division of Pulmonary and Critical Care Medicine, David Geffen School of Medicine at UCLA, Los Angeles, CA, USA; 3 Department of Pathology and Laboratory Medicine, David Geffen School of Medicine at UCLA, Los Angeles, CA, USA

**Keywords:** chemokines, neutrophils, viral infection, lung injury.

## Abstract

**Background:**

Respiratory viral infections are characterized by the infiltration of leukocytes, including activated neutrophils into the lung that can lead to sustained lung injury and potentially contribute to chronic lung disease. Specific mechanisms recruiting neutrophils to the lung during virus-induced lung inflammation and injury have not been fully elucidated. Since CXCL1 and CXCL2/3, acting through CXCR2, are potent neutrophil chemoattractants, we investigated their role in dsRNA-induced lung injury, where dsRNA (Poly IC) is a well-described synthetic agent mimicking acute viral infection.

**Methods:**

We used 6–8 week old female BALB/c mice to intratracheally inject either single-stranded (ssRNA) or double-stranded RNA (dsRNA) into the airways. The lungs were then harvested at designated timepoints to characterize the elicited chemokine response and resultant lung injury following dsRNA exposure as demonstrated qualititatively by histopathologic analysis, and quantitatively by FACS, protein, and mRNA analysis of BAL fluid and tissue samples. We then repeated the experiments by first pretreating mice with an anti-PMN or corresponding control antibody, and then subsequently pretreating a separate cohort of mice with an anti-CXCR2 or corresponding control antibody prior to dsRNA exposure.

**Results:**

Intratracheal dsRNA led to significant increases in neutrophil infiltration and lung injury in BALB/c mice at 72 h following dsRNA, but not in response to ssRNA (Poly C; control) treatment. Expression of CXCR2 ligands and CXCR2 paralleled neutrophil recruitment to the lung. Neutrophil depletion studies significantly reduced neutrophil infiltration and lung injury in response to dsRNA when mice were pretreated with an anti-PMN monoclonal Ab. Furthermore, inhibition of CXCR2 ligands/CXCR2 interaction by pretreating dsRNA-exposed mice with an anti-CXCR2 neutralizing Ab also significantly attenuated neutrophil sequestration and lung injury.

**Conclusion:**

These findings demonstrate that CXC chemokine ligand/CXCR2 biological axis is critical during the pathogenesis of dsRNA-induced lung injury relevant to acute viral infections.

## Background

Viral infections of the respiratory tract are a cause of the common cold and flu in children and adults. These infections may predispose certain patients to develop chronic respiratory disorders such as asthma, chronic obstructive pulmonary disease (COPD), pulmonary fibrosis, and bronchopulmonary dysplasia (BPD) [1]. Clinical symptoms include mucus secretion and altered airway reactivity and are hallmarked by the recruitment of inflammatory cells with resultant changes to the airway epithelial cell lining. Inflammation can also extend further into the lung to cause parenchymal disease that is characteristic of viral pneumonia as has been observed recently in severe acute respiratory syndrome (SARS) [2]. Inflammatory cell recruitment in large part is elicited by the generation of chemokines (chemotactic cytokines) that are also important in establishing a pro-inflammatory environment underlying chronic respiratory disorders such as asthma, COPD, cystic fibrosis, pulmonary fibrosis, and BPD [1,3-10].

Inflammatory changes due to viral infection result from the host immune response rather than secondary to viral replication or the viral particles themselves [11-15]. Viral infections of epithelial cells are characterized by the generation of the pro-inflammatory molecule double-stranded RNA (dsRNA) during intracellular replication of viruses. When studied in human epithelial cell lines *in vitro*, dsRNA triggers an innate immune response in host cells via generation of cytokines and chemokines involved in inflammatory cell recruitment. Specifically, dsRNA has been shown to induce activation of the neutrophil chemoattractant, interleukin-8 (IL-8/CXCL8), and regulated on activation, normal T cells expressed and secreted (RANTES) [16]. In human subjects *in vivo*, elevated tracheal IL-8/CXCL8 levels and neutrophil accumulation are found in airways of patients with asthma, COPD, and viral infection. While animal models *in vivo *have largely studied the systemic effects of intraperitoneal dsRNA treatment [17], there is a paucity of information on characterization and role of chemokines in lung inflammation and injury following intratracheal dsRNA instillation.

Murine KC/CXCL1 and MIP-2/CXCL2/3 are Glutamic acid-Leucine-Arginine-positive (ELR-positive) CXC chemokines; are structural homologs of human GRO-α/CXCL1 and GRO-β/γ/CXCL2/3, respectively; and are functional homologs of human CXC chemokines, such as IL-8/CXCL8, ENA-78/CXCL5, and GRO-α/β/γ/CXCL1/2/3 [18-21]. Both murine chemokines share the ability to signal through a G protein-coupled receptor, CXCR2 [18-20]. Their human structural and functional homologs have been associated with asthma, COPD, and viral infections of the lung [22,23].

In the current study, we hypothesized that the early inflammation and resultant lung injury from intratracheal dsRNA treatment is due, in part, to the expression of ELR-positive CXC chemokines through their interaction with their major receptor, CXCR2. To test this hypothesis, we injected dsRNA intratracheally into 6–8 week old female BALB/c mice to measure neutrophil and chemokine responses and resultant injury in the airway and lung tissue compartments. We then blocked this response by pretreating animals with antibodies to specifically neutralize neutrophil recruitment in a chemokine-dependent manner and thereby decreased lung inflammation and injury. Our animal model demonstrates the critical role of CXCR2 ligands/CXCR2 in acute lung inflammation and injury due to intratracheal dsRNA.

## Methods

### Reagents

RNA instillation: Double-stranded RNA (dsRNA, Poly IC) and single-stranded RNA (ssRNA, Poly C) were purchased from Sigma-Aldrich Corp. (St. Louis, Mo.) and reconstituted in sterile normal saline (20 μg/μl) and stored at 4°C prior to use.

#### Enzyme-linked immunoadsorption assay (ELISA) experiments

Capture and Detection antibodies to murine KC/CXCL1 and murine MIP-2/CXCL2/3 were purchased as DuoSet^® ^from R&D Systems (Minneapolis, MN).

Neutralization studies: Purified rat anti-mouse Ly-6G (Gr-1) mAb (clone RB6-8C5) was purchased from BD Pharmingen (San Diego, CA) and was used for neutrophil depletion studies as previously described [24]. Polyclonal goat anti-murine CXCR2 was produced by the immunization of a goat with a peptide containing the ligand-binding sequence Met-Gly-Glu-Phe-Lys-Val-Asp-Lys-Phe-Asn-Ile-Glu-Asp-Phe-Phe-Ser-Gly of CXCR2 [24-31]. The goat was immunized with CXCR2 in multiple intradermal sites with complete Freund's adjuvant (CFA) followed by at least 3 boosts of CXCR2 in incomplete Freund's adjuvant (IFA) as previously described. [24-31]. Direct ELISA was used to evaluate antisera titers, and sera was used for Western blot, ELISA and neutralization assays when titers had reached greater than 1/1,000,000. The CXCR2 protein sequence has been shown to contain the ligand-binding portion of the CXCR2 receptor [24-26,32]. The anti-CXCR2 antibodies have been used previously to block mouse CXCR2 *in vivo*, and has been shown to detect CXCR2 by Western blot and fluorescence-activated cell sorting analysis of neutrophils *in vivo *[24-26,32]. The anti-CXCR2 antibody has been shown to be neutralizing using both *in vitro *neutrophil chemotaxis assay and *in vivo *by abrogating the influx of neutrophils into the peritoneum of normal mice in response to exogenous ELR-positive murine CXC chemokines [24-26,32]. In vivo administration of anti-CXCR2 antibodies inhibited pulmonary neutrophil sequestration in murine models of Aspergillosis, Nocardia, and Pseudomonas pneumonia and prevented the influx of neutrophils in urine and the kidney in a murine model of Escherichia coli urinary tract infection [24-26,32]. Moreover, intraperitoneal administration of this antibody did not alter peripheral blood neutrophil counts [24-26,32]. 1 ml of antiserum against mCXCR2 and control antibody is approximately 10 mg of IgG.

### Murine model of dsRNA-induced lung injury

We used 6–8 week old female BALB/c mice to intratracheally inject either single-stranded (ssRNA) or double-stranded RNA (dsRNA) using a modification of a previously described method [30,33-36]. Mice were anesthetized with ketamine (60 mg/kg) intraperitoneally; then, under sterile conditions, the anterior neck soft tissue was dissected to expose the trachea and 50 μl RNA (20 μg/μl; 40 μg/g mouse wt) was injected via 26 gauge tuberculin needle and syringe attached to a Stepper^® ^microinjector (Indicon, Inc., Brookfield, CT) into the trachea under direct visualization. Immediately following the instillation, the skin was apposed and closed using tissue adhesive and the mice were allowed to recover from anesthesia prior to replacement into their cages.

In separate experiments, animals received either 1 ml of goat polyclonal anti-murine CXCR2, 1 ml of normal goat serum (NGS) control antibody, or 0.5 ml rat anti-mouse Ly-6G mAb or corresponding control intraperitoneally 24 hours before intratracheal injection and daily until time of sacrifice as previously described [37].

### Lung bronchoalveolar lavage and tissue preparation

At time of sacrifice, 72 h following intratracheal dsRNA or ssRNA treatment, mice were euthanized using intraperitoneal Pentobarbital (100 mg/kg) and a heparinized sample of blood was harvested. The thoracic cavity was then exposed and lungs were perfused free of blood with 1 ml 0.9% normal saline via the spontaneously beating right ventricle under constant pressure of 25 cm H_2_0. A 26 gauge butterfly needle was used to cannulate the trachea and bronchoalveolar lavage (BAL) was performed by instilling 1 ml PBS + 5 mM EDTA solution as previously described [38]. Lungs were lavaged under constant pressure of 25 cm H_2_0 and retrieved solutions were centrifuged at 900 × *g *for 15 min. The cell-free supernatants were assayed by specific ELISAs and collected cells were analyzed for total cell counts and cytospin differentials. Lung tissue was then processed for the following: calculation of lung edema; microvascular permeability; mRNA; ELISA analysis; and histopathological and immunohistochemical analysis by fixing in 4% paraformaldehyde at 25 to 30 cm H2O pressure and embedding in paraffin.

### Immunolocalization of TLR3

Paraffin-embedded tissues from dsRNA-treated and ssRNA-treated lungs were processed for immunohistochemical localization of Toll-like receptor 3 (TLR3) expression using a method previously described [33,39]. Briefly, tissue sections were dewaxed with xylene and rehydrated through graded concentrations of ethanol. Tissue nonspecific binding sites were blocked using Power Block^® ^(BioGenex, San Ramon, CA). Tissue sections were overlaid with 1:50 dilution of either control (goat) or polyclonal goat anti-TLR3 antibody (Santa Cruz Biotechnology Inc., Santa Cruz, CA). The tissue sections were washed with TRIS-buffered saline and then incubated for 60 min with secondary biotinylated antibody. The tissue sections were then washed in TRIS-buffered saline and incubated with alkaline phosphatase conjugated to streptavidin (BioGenex). Tissue sections were then incubated with Vectastain ABC reagent (Vector Laboratories, Burlingame, CA) followed by the peroxidase substrate, DAB reagent (Vector Laboratories). After optimal color development, tissue sections were immersed in sterile water, counterstained with Lerners hematoxyin, and cover slipped using an aqueous mounting solution.

### Total RNA isolation and real-time quantitative PCR

Total cellular RNA from lung tissue was isolated as previous described [30,31]. Total RNA was determined and 1 ug of total RNA was reversed transcribed into cDNA and amplified using TaqMan Gene Expression Quantification assays (Applied Biosystems (Foster City, CA) Kit 4304134). cDNA was amplified and quantified using the TaqMan 7700 Sequence Detection System and specific primers for murine CXCL1, murine CXCL2/3, murine CXCR2 and a housekeeping gene18S. The primers used were 5'-TGA-GCT-GCG-CTG-TCA-GTG-CCT-3' (sense) and 5'-AGA-AGC-CAG-CGT-TCA-CCA-GA-3' (antisense) for CXCL1 (259 bp) and 5'-GCT-GGC-CAC-CAA-CCA-CCA-GG-3' (sense) and 5'-AGC-GAG-GCA-CAT-CAG-GTA-CG-3' (antisense) for murine CXCL2/3 (359 bp). Predeveloped assay reagents (Applied Biosystems Kit 4304134) were used for murine CXCR2 and the housekeeping gene, 18S. Quantitative analysis of gene expression was done using the comparative *C*_T _(Δ*C*_T_) methods, in which *C*_T _is the threshold cycle number (the minimum number of cycles needed before the product can be detected)[40,41]. The arithmetic formula for the Δ*C*_T _method is the difference in threshold cycles for a target, (i.e., CXCR2) and an endogenous reference (i.e., housekeeping gene 18S). The amount of target normalized to an endogenous reference (i.e., CXCR2 in dsRNA-treated animals) and relative to a calibration normalized to an endogenous reference (i.e., CXCR2 in ssRNA-treated controls) is given by 2^-ΔΔCT ^[40,41]. The following is an example for comparing CXCR2 expression from dsRNA-treated animals and ssRNA-treated controls. Both CXCR2 from dsRNA-treated and ssRNA-treated controls are normalized to 18S: ΔΔ*C*_T _= Δ*C*_T _(CXCR2 expression from dsRNA-treated animals normalized to endogenous 18S)-Δ*C*_T _(CXCR2 expression from ssRNA-treated controls normalized to endogenous 18S). The calculation of 2^-ΔΔCT ^then gives a relative value when comparing the target with the calibrator, which we designate in this context as fold increase of dsRNA-treated animals to ssRNA-treated controls of the target mRNA relative quantification.

### Evans blue microvascular permeability and wet:dry analysis of lung edema

Microvascular permeability related to lung injury was measured using a modification of the Evans blue dye extravasation technique, as previously described [30,42]. Extravasation of Evans blue (Sigma-Aldrich) into the extravascular compartment was used as a quantitative measure of lung injury and changes in microvasculature permeability. Briefly, each animal received 20 mg/kg Evans blue (pH 7.34) by tail vein injection 3 h before sacrifice. At the time of sacrifice, a heparinized sample of blood was harvested, and plasma was removed by centrifugation. Six lungs from each group were perfused free of blood with 1 ml 0.9% normal saline via the spontaneously beating right ventricle and removed from the thoracic cavity. The trachea, mainstem bronchi, and surrounding mediastinal structures were removed. Evans blue was extracted from pulmonary tissues after homogenization in 1 ml of 0.9% normal saline. This volume was added to 2 vol of deionized formamide and incubated at 60C for 12 h. The supernatant was separated by centrifugation at 2000 × G for 30 min. Evans blue in the plasma and lung tissue was quantitated by dual-wavelength spectrophotometric analysis at 620 and 740 nm [43]. This method corrects the specimen's absorbance at 620 nm for the absorbance of contaminating heme pigments, using the following formula: corrected absorbance at 620 nm = actual absorbance at 620 nm – (1.426(absorbance at 740) + 0.03). We calculated a permeability index by dividing the correct pulmonary plasma Evans blue absorbance at 620 nm; this index reflects the degree of extravasation of Evans blue into the extravascular pulmonary tissue compartment.

To quantitate lung edema following dsRNA treatment, wet to dry weight ratios were obtained by ligating the lungs away from the hilum as previously described [40]. The lungs were blotted dry and weighed. They were then desiccated by incubation at 130°C overnight in a vacuum oven and re-weighed to determine their dry weight. The wet to dry ratio was then calculated.

### KC/CXCL1 and MIP-2/CXCL2/3 ELISAs

KC/CXCL1 or MIP-2/CXCL2/3 protein was quantitated using a modification of a double ligand method as previously described [30,31,40,41]. Briefly, flat-bottomed 96 well microtiter plates (Nunc Immuno-Plate I 96-F) were coated with 50 μl/well of capture antibody to murine KC/CXCL1 or MIP-2/CXCL2/3 (2 ug/ml in sterile phosphate buffered saline (PBS), for 12 hrs at room temperature and then washed with phosphate buffered saline (PBS), pH 7.5, 0.05% Tween-20 (wash buffer). Microtiter plate nonspecific binding sites were blocked with 2% BSA in PBS and incubated for 60 minutes at 37°C. Plates were washed three times with wash buffer and samples or standard were added, followed by incubation for 1 hour at 37°C. Plates were washed three times and 50 μl/well of detection antibody for murine KC/CXCL1 and MIP-2/CXCL2/3 antibodies added, and plates were incubated for 45 minutes at 37°C. Plates were washed three times, streptavidin-peroxidase conjugate (Jackson Laboratories, West Grove, PA) added, and the plates incubated for 30 minutes at 37°C. Plates were washed three times and TMB (3,3,'5,5'-tetramethylbenzidine) chromogen substrate (Kirkegaard & Perry Laboratories, Gaithersburg, MD) added. The plates were incubated at room temperature to the desired extinction, and the reaction terminated with 3 M H_2_SO_4 _solution. Plates were read at 450 nm in an automated microplate reader (Bio-Tek Instruments, Inc., Winooski, VT). Standards were 1/2-log dilutions of either KC/CXCL1 or MIP-2/CXCL2/3 from 100 ng to 1 pg/ml (50 ul/well). This ELISA method consistently detected specific chemokine concentrations in a linear fashion greater than 50 pg/ml. KC/CXCL1 and MIP-2/CXCL2/3 were specific in our sandwich ELISA without cross-reactivity to a panel of cytokines including murine C10, JE, MIP-1α, MIP-1β, human GROα, GROβ, GROγ, RANTES, and members of the CXC and CC chemokine families.

### FACS analysis of lung neutrophils

Whole lung single cell suspensions were made from harvested lungs from four mice per group using a method, as previously described [40]. Single cell suspensions (5 × 10^6 ^cells /ml) were stained with Abs: Tricolor-conjugated (BD Biosciences, Franklin Lakes, NJ) anti-murine CD45 (Caltag Laboratories, South San Francisco, CA), FITC-conjugated anti-murine MOMA-2 (macrophage surface marker; Seratec, Raleigh, NC), R-Phycoerythrin (R-PE) conjugated Rat anti-murine Ly-6G (neutrophil surface marker) and R-PE-conjugated mouse anti-murine CD3e (lymphocyte surface marker) (BD Biosciences). Dual-color-stained cell suspensions were analyzed on a FACScan flow cytometer (Becton Dickinson Immunocytometry Systems, San Jose, CA) using CellQuest 3.2.1f1 software (BD Immunocytometry Systems).

### Statistical analysis

Data were analyzed using the Microsoft^® ^Excel 2000 statistical package (Microsoft Corporation, USA). Two group comparisons were evaluated using the unpaired Students *t *test. Three group comparisons were evaluated by the ANOVA test with the post hoc analysis (i.e. Bonferroni/Dunn). Data were expressed as mean ± SEM.

## Results

### DsRNA induces expression of TLR3 on airway epithelial cells

**Figure 1:**
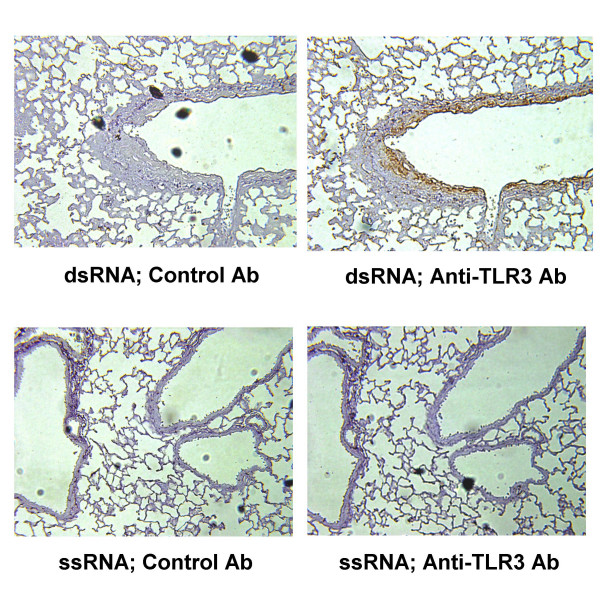
DsRNA induces expression of TLR3 on airway epithelial cells. Immunohistochemistry of lungs at 72 h following ssRNA or dsRNA treatment. Slides were stained with either control goat IgG or anti-TLR3 Ab (100X) (*n *= 4 mice per group).

Since *in vitro *studies in human epithelial cells have demonstrated that dsRNA induces the generation of chemokines involved in leukocyte recruitment, we performed *in vivo *studies using a murine model system of intratracheal dsRNA-induced inflammation and injury to mimic an acute viral infection and thus dissect the mechanisms related to this process. The putative receptor for dsRNA has been identified as Toll-like receptor 3 (TLR3), thus we first determined whether dsRNA treatment was associated with expression of TLR3. Immunolocalization using goat anti-murine TLR3 Ab showed markedly increased expression of TLR3 localized to the surface airway epithelium of dsRNA-treated lungs as compared to ssRNA-treated controls at 72 h. Specificity to TLR3 was demonstrated by lack of staining on control goat IgG-stained sections from both dsRNA and ssRNA-treated groups (Fig. 1).

### DsRNA induces lung neutrophil infiltration

**Figure 2:**
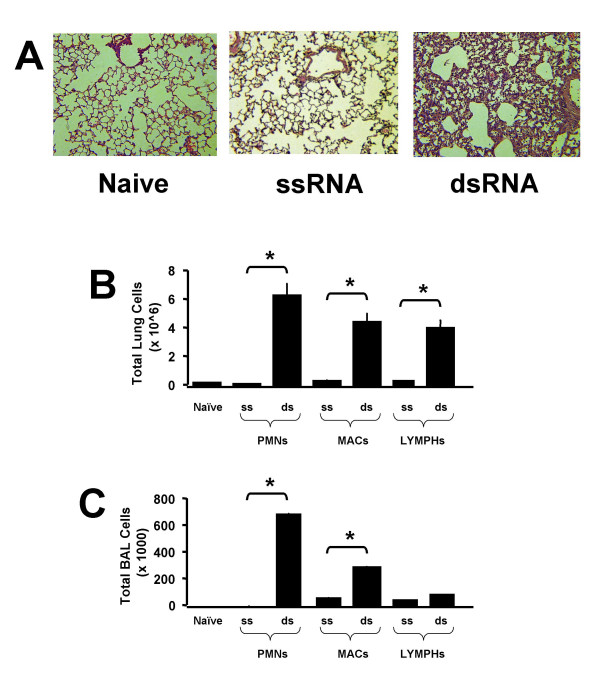
DsRNA induces lung neutrophil infiltration. **(A) **Histopathology of naïve lung and at 72 h following treatment with ssRNA or dsRNA. Representative photomicrographs with H & E staining (100X; *n *= 4 mice per group). **(B) **Total lung cells via FACS analysis of whole-lung single-cell suspensions at 72 h (*n *= 4 mice per group; *p < 0.05). **(C) **Total cells via BAL fluid cell count at 72 h (*n *= 4 mice per group; *p < 0.05).

Mice treated with intratracheal dsRNA were noted to have significant intraparenchymal and airway leukocyte infiltration at 72 h following treatment as compared to naïve and ssRNA-treated controls as demonstrated by histopathology (Fig. 2A). No significant differences in cellular infiltrates were noted among the histopathologic groups at earlier timepoints 4, 12, 24, and 48 h following dsRNA treatment (data not shown). FACS and BAL analysis at 72 h following dsRNA treatment specifically showed that dsRNA induced significant neutrophil recruitment compared to controls (Fig. 2B and 2C).

### DsRNA induces increased lung injury

**Figure 3:**
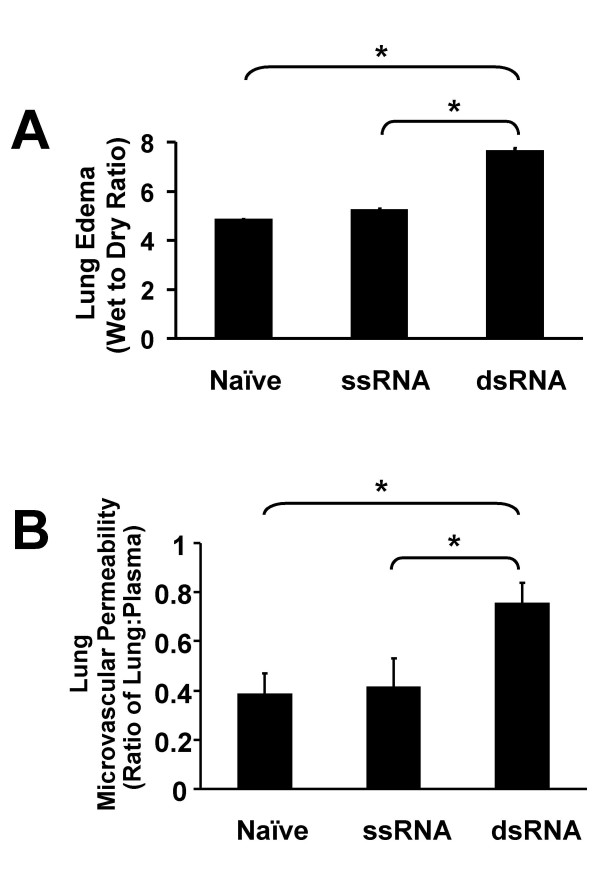
DsRNA induces lung injury. **(A) **Wet:Dry ratio at 72 h (*n *= 6 mice per group; *p < 0.05). **(B) **Evans blue permeability index at 72 h (*n *= 6 mice per group; *p < 0.05).

To determine if the influx of neutrophils into lung airways and parenchyma results in lung injury, we measured two markers of lung injury to quantify changes in lung edema and lung vascular permeability. Results showed a significant increase in the wet:dry ratio in dsRNA-treated lungs as compared to controls at 72 h following dsRNA treatment (Fig. 3A). Similarly, measurement of the lung:plasma extravasation ratio of Evans blue dye also showed a significant increase in microvascular permeability in dsRNA-treated lungs as compared to controls (Fig. 3B).

### Neutrophil depletion decreases dsRNA-induced lung inflammation and injury

**Figure 4:**
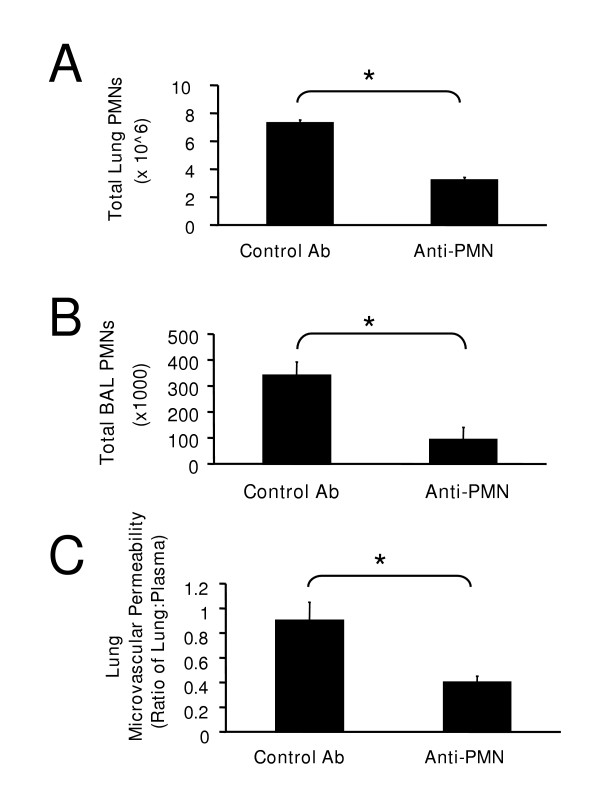
Neutrophil depletion decreases dsRNA-induced lung inflammation and injury. **(A) **Total lung PMNs via FACS analysis of whole-lung single-cell suspensions at 72 h. (*n *= 4 mice per group; *p < 0.05). **(B) **Total PMNs via BAL fluid cell count at 72 h (*n *= 4 mice per group; *p < 0.05). **(C) **Evans blue permeability index at 72 h (*n *= 6 mice per group; *p *< 0.05). Mice were pretreated control Ab or anti-PMN Ab at times -24, 0, 24, and 48 h following IT dsRNA treatment.

With the finding that an increase in lung neutrophils coincided with an increase in lung injury in dsRNA-treated lungs, we next attempted to determine if this was a causal relationship by inducing neutropenia and measuring changes in lung neutrophil influx and lung injury. Mice were passively immunized with specific anti-mouse Ly-6G mAb or corresponding control at -24 h as well as 0, 24, and 48 h following dsRNA treatment. Lungs were harvested at 72 h and results showed that animals pretreated with neutrophil depleting mAb had a significant decrease in total neutrophil counts in both lung tissue and airways as compared to controls as reflected by FACS analysis and BAL (Fig. 4A and 4B). Importantly, BAL samples of animals pretreated with anti-mouse Ly-6G mAb antibody showed a specific reduction in neutrophil number but no reduction in monocyte numbers (data not shown). Furthermore, neutrophil depletion resulted in decreased lung microvascular permeability back to baseline values (Fig. 4C).

### DsRNA induces elevated KC/CXCL1 and MIP-2/CXCL2/3 mRNA and protein levels

**Figure 5:**
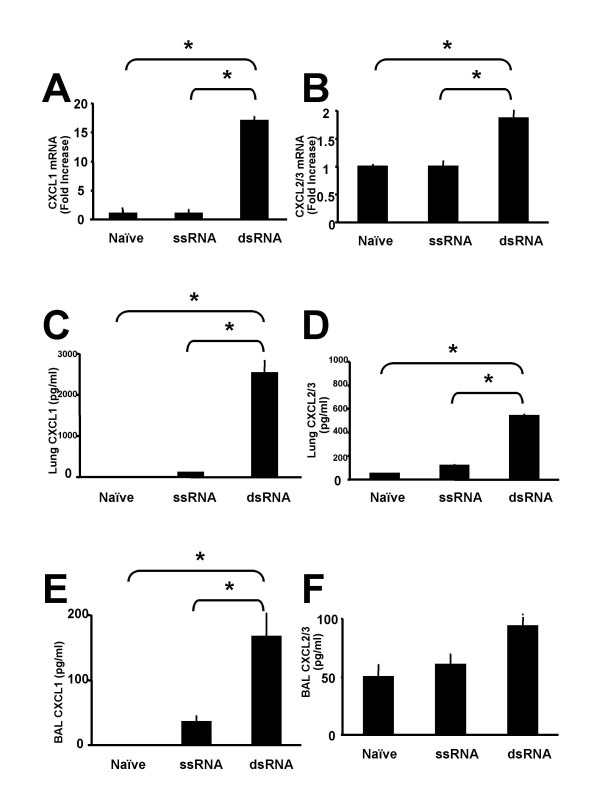
DsRNA induces elevated KC/CXCL1 and MIP-2/CXCL2/3 mRNA and protein levels. **(A and B) **Quantitative levels of CXCL1 and CXCL2/3 mRNA, respectively, in naïve lung and at 72 h following treatment with ssRNA or dsRNA (*n *= 6 mice per group; *p < 0.05). **(C and D) **Quantitative levels of CXCL1 and CXCL2/3 protein, respectively, in the lungs at 72 h (*n *= 6 mice per group; *p < 0.05). **(E and F) **Quantitative levels of CXCL1 and CXCL2/3 protein, respectively, in BAL fluid at 72 h (*n *= 6 mice per group; *p < 0.05).

Since neutrophil influx was shown to result in lung injury in dsRNA-treated lungs, we next identified which specific factors, such as chemokines, were responsible for neutrophil recruitment. We focused on the ELR+ chemokines KC/CXCL1 and MIP-2/CXCL2/3, which are known to have neutrophil chemoattractant properties. DsRNA treatment resulted in significant increases in lung KC/CXCL1 mRNA levels as well as in protein levels from whole lung homogenates and BAL when compared to controls (Fig. 5A, C, and 5E) at 72 h following dsRNA treatment. Similarly, mRNA levels and lung protein expression of MIP-2/CXCL2/3 were also significantly elevated with an increasing trend noted in BAL protein from dsRNA-treated lungs as compared to controls (Fig. 5B, D, and 5F). Levels of CXCR2 chemokine ligands at earlier timepoints showed only a small increase (two-fold) in the induction of KC/CXCL1 and MIP-2/CXCL2/3 as early as 4 h following dsRNA-exposure (data not shown), but the maximal increase (>ten-fold for KC/CXCL1 and >four-fold for MIP-2/CXCL2/3) occurred at 72 h following dsRNA-exposure.

### DsRNA increases CXCR2 expression in lungs

**Figure 6:**
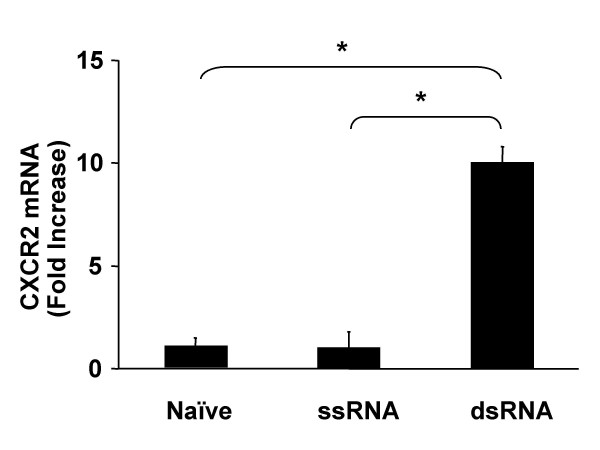
DsRNA increases CXCR2 expression in lungs. Quantitative real-time PCR was determined by TaqMan analysis for CXCR2 mRNA from naïve lung and at 72 h following ssRNA or dsRNA treatment (*n *= 6 mice per group; *p < 0.05).

CXCR2 is the shared cellular receptor for the murine CXC chemokine ligands KC/CXCL1 and MIP-2/CXCL2/3 [18-20]. The finding of increased levels of KC/CXCL1 and MIP-2/CXCL2/3 associated with dsRNA-induced neutrophil sequestration and lung injury led us to evaluate the expression of CXCR2 mRNA in the lungs of these animals. Lung homogenates from dsRNA-treated animals had significantly increased CXCR2 mRNA expression as compared to controls (Fig. 6). The expression of CXCR2 mRNA paralleled its ligand expression, neutrophil sequestration, and lung injury at 72 h following dsRNA treatment (Figs. 2, 3, and 5).

### Inhibition of CXCR2 inhibits infiltration of neutrophils and attenuates dsRNA-induced lung injury

**Figure 7:**
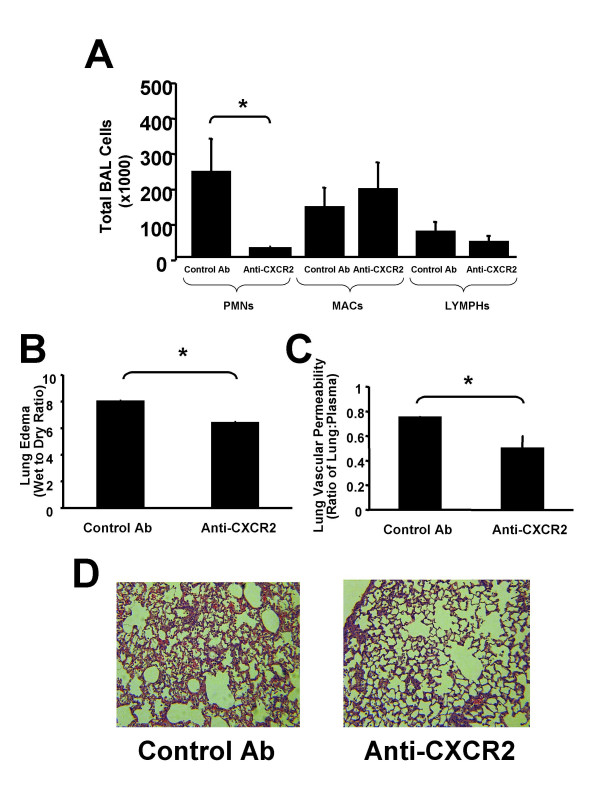
Inhibition of CXCR2 inhibits dsRNA-induced neutrophil recruitment and attenuates dsRNA-induced lung injury. **(A) **Total cells via BAL fluid cell count at 72 h (*n *= 4; *p < 0.05). **(B) **Wet:Dry ratio at 72 h (*n *= 6 mice per group; *p < 0.05). **(C) **Evans blue permeability index at 72 h (*n *= 6 mice per group; *p < 0.05). **(D) **Histopathology of lungs at 72 h following dsRNA treatment. Representative photomicrographs with H & E staining (100X; *n *= 4 mice per group). Mice were pretreated with normal goat serum (control Ab) or anti-CXCR2 Ab at times -24, 0, 24, and 48 h following IT dsRNA treatment.

To better understand the mechanism partly responsible for dsRNA-induced lung neutrophilia and injury, we determined whether inhibiting CXCR2 ligand interaction with CXCR2 significantly decreased neutrophil recruitment during the pathogenesis of dsRNA-induced lung injury. Mice were passively immunized with specific neutralizing anti-murine CXCR2 or with control antibody at -24 h, as well as 0, 24, and 48 h following dsRNA treatment. Lungs were harvested at 72 h and results showed that BAL neutrophil counts from animals that received anti-CXCR2 Ab were significantly reduced as compared to control animals that received normal goat serum (Fig. 7A). Furthermore, measurement of lung edema and lung microvascular permeability also showed significant decreases in wet:dry and Evans blue extravasation in mice treated with anti-CXCR2 compared to NGS-treated controls (Fig. 7B and 7C). Finally, histopathologic comparison of lung fields from anti-CXCR2 pretreated mice showed marked reduction in leukocytic infiltrate as compared to NGS-pretreated controls (Fig. 7D).

## Discussion

Respiratory viral infections are characterized by a two-component immune response comprised of an innate component that is fully functional before viral entry into the epithelium and an adaptive component that develops in response to the continued presence of the virus [1]. The innate or acute inflammatory component is associated with a predominance of infiltrating neutrophils. While many viral infections of the lung are self-limiting, the associated lung injury due to this initial event may be critical in establishing a pro-inflammatory environment underlying certain chronic respiratory disorders such as asthma, COPD, cystic fibrosis, pulmonary fibrosis, viral pneumonia, and bronchopulmonary dysplasia [1,3-10]. The host's inflammatory response to viral replication leads to pulmonary pathology hallmarked by leukocyte infiltrate and resultant microvascular leak that progresses to lung edema and the clinical signs of pneumonia. As the lung injury persists or recurs with multiple subsequent viral infections, pulmonary vascular and airway remodeling may occur and eventually lead to development of airway hyper-reactivity and/or interstitial fibrosis [44]. Viral infections are mediated by dsRNA, a proinflammatory molecule generated during viral replication. DsRNA binds to its cell surface receptor, TLR3, and activates the production of downstream gene products, such as CXC chemokines. In this study, we hypothesized that the interaction between CXCR2 and ELR-positive CXC chemokines expressed during dsRNA-induced lung inflammation is critical in mediating neutrophil recruitment, a pivotal process required for dsRNA induced lung injury in viral infections.

Previous studies have demonstrated that mice exposed to a live virus via intranasal inoculation generate a systemic acute-phase response with maximal pulmonary chemokine response at one week that includes both CC and CXC chemokines and is mouse strain-dependent (response in BALB/c greater than in C57BL/6 mice) [45,46]. A similar study using intratracheal delivery of live virus also demonstrated marked pulmonary pathology including mucous cell metaplasia and airway epithelial remodeling [47]. Another study focusing specifically on the effects of intratracheal dsRNA at a low dose found similar systemic inflammatory effects but specific pulmonary effects only when dsRNA was delivered in conjunction with IFNγ [48]. Finally, a recent study examined the effects of inhibiting the CC chemokine receptor, CCR1, during live-virus exposure in mice and showed that mortality during pneumovirus infection was decreased [49]. The present study extends these findings by first determining the effects of high-dose intratracheal dsRNA alone on neutrophil recruitment and lung injury in BALB/c mice and then subsequently blocking these effects via inhibition of the CXC chemokine receptor, CXCR2.

To determine the effects of dsRNA *in vivo*, we first characterized our murine model by performing a time-course of dsRNA to observe histopathologic changes at 0, 4, 12, 24, 48 and 72 h following intratracheal dsRNA delivery. We used a maximal dose of dsRNA at 40 μg/g mouse wt after initial studies at lower doses (4 μg/g and 20 μg/g) showed minimal leukocytic infiltrate. Furthermore, we are aware of only one other publication that describes intratracheal dsRNA delivery that showed no effects when used alone at low concentration (10 ug/g) [48]. Histopathological analysis demonstrated a significantly increased leukocytic infiltrate at 72 h following intratracheal dsRNA as compared to earlier time points. We thus chose this observation as the basis to focus upon the 72 h timepoint. Other studies using administration of dsRNA in BALB/c mice *in vivo *have also shown a maximal innate immune response starting at 72 h following dsRNA exposure [50] Further characterization at 72 h following intratracheal dsRNA showed that neutrophils were a predominant cell type and that there was an associated injury to alveolar-capillary membrane integrity as shown by increased lung edema and microvascular leak.

Having characterized the histopathological damage caused by intratracheal dsRNA, we then focused on the underlying mechanisms responsible for promoting the inflammation and subsequent lung injury. Our findings of a significant increase in neutrophil infiltration following dsRNA treatment are consistent with findings from previous studies using a live virus that also resulted in early neutrophil infiltration [47]. In order to determine whether neutrophil influx was causally linked to the lung injury observed in our system, we performed studies using a monoclonal antibody to the Ly6G antigen on the surface of mouse granulocytes to specifically deplete neutrophils. These results showed decreased numbers of lung neutrophils by FACS analysis and BAL as expected with no reduction in monocyte number, and also showed a decrease in lung microvascular leak and therefore decreased lung injury associated with decreased neutrophil recruitment. However, the molecular and cellular mechanisms involved in recruiting these neutrophils remained to be fully elucidated.

Elegant *in vitro *studies have demonstrated that dsRNA can induce IL-8 expression from human bronchial epithelial cells [16]. Furthermore, *in vivo *studies using live virus have also demonstrated that CXC chemokines are generated during lung inflammation [45-47]. Having demonstrated that lung injury due to intratracheal dsRNA is dependent on neutrophil infiltration, we determined that CXCL1 and CXCL2/3 expression was significantly greater in the lungs of dsRNA-treated mice than in lungs of ssRNA-treated controls. Furthermore, expression of CXCR2 mRNA was similarly increased in parallel to the production of both CXCL1 and CXCL2/3 ligands and neutrophil sequestration during dsRNA-induced lung inflammation. Other studies of inflammatory diseases, such as ventilator-induced lung injury, pneumonia, and hyperoxia-induced lung injury have demonstrated the importance of CXCR2 expression and its role in neutrophil recruitment during the pathogenesis of these diseases [24,40,41,51]. Collectively, these studies demonstrate that augmented levels of CXCR2 ligands are important in the recruitment of neutrophils during the pathogenesis of inflammatory diseases and suggest that the interaction between CXCR2 ligands and CXCR2 may be pivotal in the recruitment of neutrophils to the lung during dsRNA-induced lung injury.

Based on these findings, we performed proof of principle studies *in vivo *using an antibody-mediated neutralization strategy to evaluate the direct role for CXCL1 and CXCL2/3 ligands and their interaction with CXCR2 during the pathogenesis of dsRNA-induced lung injury. The anti-CXCR2 Ab-treated mice demonstrated significant reductions specific toneutrophil infiltration that were paralleled by a decrease in lung injury. This suggests that if other leukocytes are recruited there are redundant pathways involved in their recruitment that are CXCR2-independent. Our findings are similar to previous neutralization studies in virus-infected mice that showed functional antagonism to the chemokine receptor CCR1 reduced mortality [49]. Our study is the first to demonstrate that neutralization of CXCR2 results in decreased neutrophil recruitment and lung injury induced by intratracheal dsRNA.

TLR3 is a pattern-recognition cell surface receptor that is responsible for specifically recognizing extracellular dsRNA [52]. It is a member of a family of toll-like receptors (TLRs) that aid the host in combating infections when microbial pathogens bind to their specific toll-like receptor to trigger NFκB and downstream generation of cytokines and costimulatory molecules during the innate immune attack. TLR3 thus plays an important role in host defense against viral infections. Our study showed that dsRNA treatment induced the expression of TLR3 on the cell surface of airways as compared to ssRNA-treated controls animals. The upregulation of TLR3 in dsRNA-exposed mouse airways suggests a positive feedback system in which epithelial cells exposed to dsRNA increase TLR3 expression on their cell surface. A similar upregulation of TLR3 has previously been described in epithelial cells *in vitro *treated with Respiratory syncytial virus (RSV)[53]. These findings are also consistent with *in vivo *studies using monoclonal antibody to TLR3 and genetic knockout mice of TLR3 [52,54] that establish the role of TLR3 in dsRNA recognition and raise the interesting possibility that TLR3 may be involved in the signaling pathway mediating dsRNA induction of CXC chemokines.

## Conclusion

In conclusion, we have demonstrated that the biological axis of CXCR2 ligand/CXCR2 signaling plays a pivotal role in mediating neutrophil recruitment and dsRNA-induced lung injury. This mechanism may be critical for the promotion of further lung injury and remodeling in patients who eventually develop chronic lung diseases such as asthma, COPD, cystic fibrosis, pulmonary fibrosis or bronchopulmonary dysplasia. The findings of the current study support the contention that CXCR2 ligands and CXCR2 mediate dsRNA-induced lung injury, and may be a therapeutic target to attenuate this pathology.

## Competing interests

The author(s) declare that they have no competing interests.

## Authors' Contributions

VL designed and carried out all experiments and drafted the manuscript; JB and MK contributed to analysis and interpretation of data; MB and YX contributed to performing animal experiments; and RS provided final analysis and interpretation of data.
